# Counteracting Age-related Loss of Skeletal Muscle Mass: a clinical and ethnological trial on the role of protein supplementation and training load (CALM Intervention Study): study protocol for a randomized controlled trial

**DOI:** 10.1186/s13063-016-1512-0

**Published:** 2016-08-09

**Authors:** Rasmus Leidesdorff Bechshøft, Søren Reitelseder, Grith Højfeldt, Josué Leonardo Castro-Mejía, Bekzod Khakimov, Hajar Fauzan Bin Ahmad, Michael Kjær, Søren Balling Engelsen, Susanne Margrete Bølling Johansen, Morten Arendt Rasmussen, Aske Juul Lassen, Tenna Jensen, Nina Beyer, Anja Serena, Frederico Jose Armando Perez-Cueto, Dennis Sandris Nielsen, Astrid Pernille Jespersen, Lars Holm

**Affiliations:** 1 Institute of Sports Medicine Copenhagen, Department of Orthopedic Surgery M, Bispebjerg Hospital, Bispebjerg Bakke 23, DK-2400 Copenhagen, NV Denmark; 2 Department of Biomedical Sciences, Faculty of Health and Medical Sciences, University of Copenhagen, Copenhagen, Denmark; 3 Department of Food Science, Faculty of Science, University of Copenhagen, Copenhagen, Denmark; 4 Copenhagen Prospective Studies on Asthma in Childhood, Faculty of Health and Medical Sciences, University of Copenhagen, Copenhagen, Denmark; 5 Danish Pediatric Asthma Center, Gentofte Hospital, University of Copenhagen, Copenhagen, Denmark; 6 SAXO Institute, Faculty of Humanities, University of Copenhagen, Copenhagen, Denmark; 7 Musculoskeletal Rehabilitation Research Unit, Department of Physical and Occupational Therapy, Bispebjerg Hospital, Copenhagen, Denmark; 8 Arla Foods Ingredients Group P/S, Viby J, Denmark

**Keywords:** Elderly, Protein, Whey, Muscle, Strength training, Gut microbiome, Plasma metabolome

## Abstract

**Background:**

Aging is associated with decreased muscle mass and functional capacity, which in turn decrease quality of life. The number of citizens over the age of 65 years in the Western world will increase by 50 % over the next four decades, and this demographic shift brings forth new challenges at both societal and individual levels. Only a few longitudinal studies have been reported, but whey protein supplementation seems to improve muscle mass and function, and its combination with heavy strength training appears even more effective. However, heavy resistance training may reduce adherence to training, thereby attenuating the overall benefits of training. We hypothesize that light load resistance training is more efficient when both adherence and physical improvement are considered longitudinally. We launched the interdisciplinary project on Counteracting Age-related Loss of Skeletal Muscle Mass (CALM) to investigate the impact of lifestyle changes on physical and functional outcomes as well as everyday practices and habits in a qualitative context.

**Methods:**

We will randomize 205 participants older than 65 years to be given 1 year of two daily nutrient supplements with 10 g of sucrose and 20 g of either collagen protein, carbohydrates, or whey. Further, two groups will perform either heavy progressive resistance training or light load training on top of the whey supplement.

**Discussion:**

The primary outcome of the CALM Intervention Study is the change in thigh cross-sectional area. Moreover, we will evaluate changes in physical performance, muscle fiber type and acute anabolic response to whey protein ingestion, sensory adaptation, gut microbiome, and a range of other measures, combined with questionnaires on life quality and qualitative interviews with selected subjects. The CALM Intervention Study will generate scientific evidence and recommendations to counteract age-related loss of skeletal muscle mass in elderly individuals.

**Trial registration:**

ClinicalTrials.gov NCT02034760. Registered on 10 January 2014.

ClinicalTrials.gov NCT02115698. Registered on 14 April 2014.

Danish regional committee of the Capital Region H-4-2013-070. Registered on 4 July 2013.

Danish Data Protection Agency 2012-58-0004 – BBH-2015-001 I-Suite 03432. Registered on 9 January 2015.

## Background

On the basis of demographic extrapolations, the number of elderly citizens above the age of 65 years will increase in the next three decades by 50–200 %, with the specific proportion being dependent on country [1, 2]. With biological aging, physical function often decreases. This increases the risk of developing frailty [3], indicating higher risk for adverse events. Even in healthy, active, and independently living older people, a gradual loss of muscle mass, termed *sarcopenia* [4], takes place at an annual rate of up to 1–2 % starting in the sixth decade of life [5–7]. The loss of muscle mass is accompanied by an even faster deterioration of muscle strength of up to 3.5 % per year [8]. If allowed to progress, the development of sarcopenia is associated with increased risk of falling [9], decreased satisfaction with life [10], and even an increased mortality rate [11]. The impact of sarcopenic progression may become detrimental to an individual’s personal life and autonomy, and the societal implications are vast when one considers future healthcare and nursing expenditures. Therefore, in the present study, we aim to test the efficacy and feasibility of different strategies for counteracting muscular deterioration. Preferably, these strategies should be easy to integrate into everyday life for the majority of the aging population to induce maximal efficiency at both individual and societal levels.

Previous research has described the multifactorial nature of the development of sarcopenia [12–14]. On the basis of the aging muscle becoming less sensitive to daily anabolic stimuli due to protein intake [15] and muscular activity [16, 17], it is suggested that exactly these two factors possess a high potential to antagonize sarcopenia. Further, the nutrition and exercise training strategies are self-manageable, allowing a certain degree of flexibility for adjustment to personal preferences and everyday practices. Although their mutual dependency is acknowledged [18, 19], the “dosing” of each factor remains questionable, and the impact during long-term exposure is unknown. In contrast to studies of strategies used for treating individuals who already have loss of muscle mass and function, the aim of the Counteracting Age-related Loss of Skeletal Muscle Mass (CALM) Intervention Study is to evaluate strategies to prevent sarcopenic progression in healthy, independently living aging individuals.

With regard to protein intake, cohort studies strongly suggest an association between high protein intake and decreased rates of age-dependent decline in physical performance and reduced risk of frailty [20–22]. However, researchers in intervention studies have reported inconclusive effects on physical function and muscle mass after administering protein supplements for longer periods [23–25]. Research has shown that ingestion of 10 g of essential amino acids [26], corresponding to roughly 20 g of whey protein or even 35–40 g of dairy protein at rest [27] or after exercise [28], can stimulate muscle protein synthesis (MPS) fully. The quality of the ingested protein is a matter of concern, and the protein digestibility-corrected amino acid score (PDCAAS) [29] is a way to evaluate this. Protein amino acid composition and protein digestibility are the two notable factors that determine the PDCAAS, but using hydrolyzed protein maximizes protein digestibility, especially for proteins with slow digestibility, such as casein, leaving amino acid composition as the most important factor for the protein of choice when investigating the effect on muscle mass. Research has shown that whey protein appears advantageous in comparison with other protein sources [30, 31]. While the focus has been mainly on protein type and quantity, experimental results combined with eating patterns of elderly persons recently led to the hypothesis that intake distribution may be of importance [32]. In Denmark, calculations suggest that citizens between 65 and 75 years of age consume a mean of 1.1 g of protein per kilogram of body weight per day [33, 34], indicating that the average senior citizen consumes a reasonable amount of protein [13, 35–37]. This amount is well above the present recommended dietary allowance of 0.8 g/kg body weight [29, 38], which is based on the estimated average requirements ±2 SD. The pattern of protein ingestion is not currently mentioned in recommendations, and data derived from elderly citizens in the United States [39] and Denmark (Fagt S, Danskernes Kostvaner 2011-2013, unpublished data) demonstrate that almost half of the daily amount of protein is ingested at dinner. The implications of this may be that the MPS is maximally stimulated only once daily [27, 40, 41], despite daily average protein consumption appearing to be adequate. Therefore, we hypothesize that a recommendation for enhanced daily protein intake should include a guideline for an evenly distributed protein intake throughout the day to optimize muscle protein accretion [42, 43]. Promoting a healthier eating pattern may be successful in improving food choices, but this has a modest success in terms of actual lifestyle changes [44]. Therefore, if research should convert any new knowledge on the timely provision of additional protein intake into actual changes in daily eating patterns and life routines in the elderly target group, scientists must take into account the short-term (weeks) and long-term (months) acceptability of daily ingestion of food supplements. Additionally, to describe the impact of daily supplementation with protein on general health, researchers should also investigate additional benefits or compromising side effects of the protein intake.

**Fig. 1:**
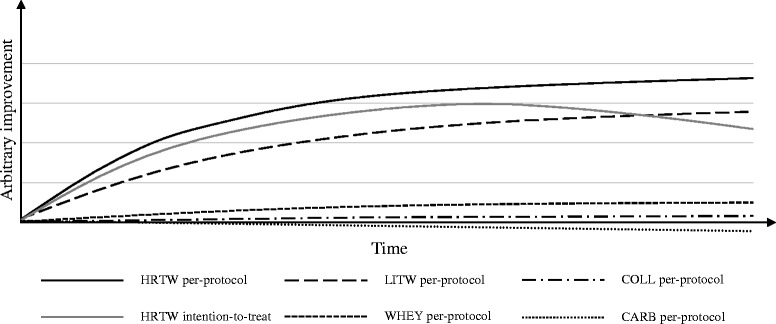
Hypothesized improvements over time with different interventions. *Black lines* mark the expected effect of per-protocol analysis: HRTW (*solid line*), LITW (*long*-*dashed line*), WHEY (*short-dashed line*), COLL (*dashed-dotted line*), and CARB (*dotted line*) interventions when analyzed per protocol. The *gray line* marks the expected effect intention-to-treat analysis of HRTW. *CARB* carbohydrate supplementation group, *COLL* collagen supplementation group, *HRTW* heavy resistance training with whey supplementation, *LITW* light-intensity training with whey supplementation, *WHEY* whey supplementation

Heavy load muscle training is undoubtedly the most effective exercise strategy to gain muscle mass and strength [45–48], and even very old individuals can benefit from training by experiencing an increase in muscle mass and strength [18, 19, 49–52]. However, at a societal level, the overall impact of implementing a certain training regime reflects a balance between the stimulatory effect of exercise training per se and tolerability and adherence to the training modality and setting. Activities of more moderate intensity, preferably those that are simple, convenient, and cheap [53], are associated with increased participation among the elderly. Further, these activities should be performed in an informal setting [54], and sessions in a training program seeking high adherence should be either home-based or conducted in smaller groups with peers [55, 56]. Therefore, despite an increased training effect per se, the feasibility of heavy load strength training requiring admittance to a fitness center or similar facility may be questionable for the elderly population [57]. As demonstrated in Fig. 1, home-based light load resistance training may prove to be a better recommendation for preventing age-related decreases in muscle mass and physical function because of increased adherence, but longitudinal research in the elderly is lacking.

**Fig. 2:**
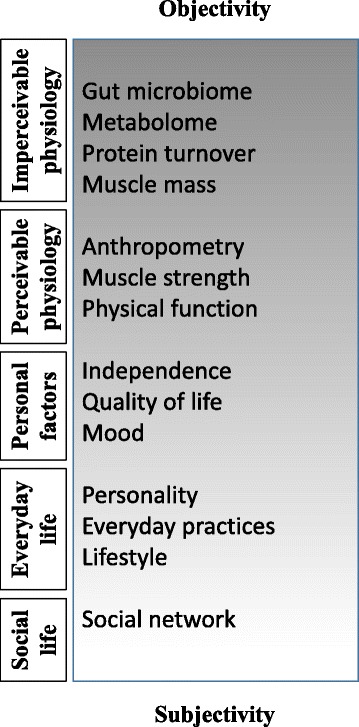
Spectrum of outcome variables. In the interdisciplinary Counteracting Age-related Loss of Skeletal Muscle Mass Intervention Study, we are investigating the entire spectrum of possible impacts of the intervention by applying objective, quantitative measures of the body and subjective, qualitative investigations of the participants

We have launched a 1-year longitudinal randomized controlled trial (RCT), partly single-blinded/partly double-blinded, to investigate the impact of protein quality and training intensity on different variables. The primary outcome measure is the change in knee extensor skeletal muscle cross-sectional area (CSA) over the course of 1 year of intervention. The secondary and tertiary outcomes are listed in the Measurements section below. In this RCT, we are integrating the evaluation of the interventions’ impact on general health, physical and functional parameters, and impact on daily life routines and practices as well as on the acceptability of the interventions, covering the entire spectrum from objective physiological measures to subjective investigations on the participants. All results will be fed into the integrative and interdisciplinary outcomes of the CALM Intervention Study (Fig. 2).

### Working hypotheses

We designed the CALM Intervention Study to investigate two overarching working hypotheses:

1. Whey protein hydrolysate supplementation is more efficient than collagen protein hydrolysate and carbohydrate in improving muscle size when administered to healthy elderly individuals.

2. With whey protein hydrolysate supplementation, the addition of center-based heavy resistance training is advantageous to home-based light load resistance training, and both of these regimens are superior to no training at all, in regard to improving muscle mass, overall health, and physiological parameters only with a per-protocol analysis. This will not be the case in regard to intention-to-treat analysis, where a markedly higher adherence is presumed in the home-based training group, leading to a similar or superior response compared to the center-based heavy resistance training group.

## Methods/design

### Overview

The CALM Intervention Study is a single-center, partly single- and partly double-blinded, multidisciplinary RCT. Collaborators are affiliated with (1) the Faculty of Science, Department of Food Science, (2) the Faculty of Humanities, SAXO Institute, and (3) the Faculty of Health and Medical Sciences, Department of Biomedical Sciences, all at the University of Copenhagen and the Institute of Sports Medicine Copenhagen, Bispebjerg Hospital, Copenhagen, Denmark. We wrote the protocol using the Standard Protocol Items: Recommendations for Interventional Trials (SPIRIT) guidelines for clinical trial protocols (http://www.spirit-statement.org/spirit-statement) and registered the protocol at ClinicalTrials.gov. Any changes will be reported there and to the local ethics committee.

### Recruitment of participants

The CALM Intervention Study will include 205 participants living in the Greater Copenhagen area through local newspapers, magazines, radio programs, social media, and presentations at senior centers and public events. See the Statistics and power calculations section below for further explanation on recruitment requirements. Recruitment commenced in January 2014. Exclusion criteria are listed in Table 1.

**Table 1:** Exclusion criteria

	Criteria
1	Care dependency
2	Disability in lower extremities
3	Arthritis or arthrosis in knee or hip joints, arthritis requiring medication, or other rheumatic diseases potentially affecting joints or muscles
4	Diagnosed or suspected knee osteoarthritis (based on EULAR criteria: three symptoms and three signs); excluded if more than one of the following thee symptoms are found: morning stiffness <30 minutes, persistent knee pain, or functional limitations
5	Bilateral knee alloplastic and hip alloplastic material
6	Connective tissue disorders
7	Severe COPD (FEV_1_/FVC ratio <70 % and FEV_1_ < 50 % of predicted value (GOLD stage 3 or 4)
8	Unstable cardiac arrhythmias or decreased LVEF (<60 %)
9	Gut diseases affecting food absorption
10	Surgical diseases affecting ability to conduct heavy load strength exercise
11	Embodied magnetic metal
12	Endocrinological diseases potentially affecting muscles (diabetes mellitus, growth hormone-treated, sex hormone-treated, or untreated thyroid diseases)
13	Alcohol consumption >21 U/week for men and 14 U/week for women (1 U = 15.2 ml of alcohol)
14	Participation in studies using the same stable isotopically labeled tracers as this study (i.e., L-[ring-^13^C_6_]phenylalanine) within the last 6 months
15	Medications: systemic corticosteroids, sex hormone therapy, anti-sex hormone therapy, anticoagulants (thrombin inhibitors, K-vitamin antagonists, heparins, pentasaccharides, factor Xa inhibitors, thrombocyte inhibitors except nonsteroidal anti-inflammatory drugs and acetylsalicylic acid).
16	>1 h of weekly heavy strength training
17	Dementia or other severe cognitive impairment
18	Not holding Danish citizenship or not fluent in Danish

*Abbreviations: COPD* chronic obstructive pulmonary disease, *EULAR* European League Against Rheumatism, *FEV*_*1*_ forced expiratory volume in 1 second, *FVC* forced vital capacity, *GOLD* Global Initiative for Chronic Obstructive Lung Disease, *LVEF* left ventricular ejection fraction

### Information and screening

Upon first contact, a researcher informs the possible participants briefly about the project and sends written information by mail or e-mail. If the candidate is still interested after having read the material, the staff member subsequently screens for exclusion criteria and invites the person to an information meeting after which participants give their written informed consent in accordance with Declaration of Helsinki II. The protocol has been approved by The Danish Regional Committees of the Capital Region on 4 July 2013 (number H-4-2013-070).

At the information meeting, a medical member of the research team delivers the information verbally followed by a health screening including blood pressure, blood samples, and heart auscultation to determine if the candidate can safely complete the interventions. Then, the participant performs a 30-second chair-stand test that we use for stratified randomization only.

### Randomization

**Fig. 3:**
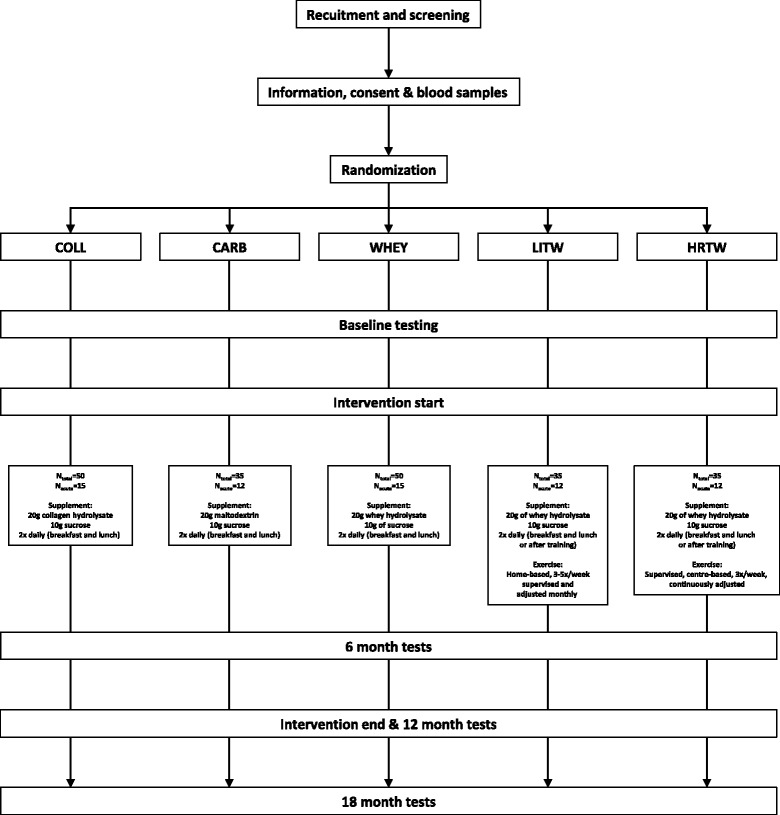
Participant flow. N_total_ represents the expected number of inclusions in each group. N_acute_ represents the expected number of participants who will complete the measurements of fractional synthesis rate at 0 and 12 months. *COLL* Collagen supplementation, *CARB* Carbohydrate supplementation, *WHEY* Whey supplementation, *LITW* Light intensity resistance training and whey supplementation, *HRTW* Heavy resistance training and whey supplementation

Following screening and health examinations, we enroll candidate participants and researchers who are not involved in interventions or tests that are sensitive to blinding randomize them using the open-source minimization program MinimPy 0.3 [58, 59]. To ensure even distribution of subjects throughout the study period, we decided to employ a stratified, biased coin minimization with 0.95 base probability and to use allocation ratios corresponding to the group sizes (see Statistics and power calculations section below). Participant stratification is based on a 30-second chair-stand test (<16 or ≥16) and on gender to ensure equal allocation to one of the five intervention groups and further to determine if the participant will participate in invasive acute mechanistic MPS trials (see Muscle protein synthesis section below), as demonstrated in Fig. 3.

### Interventions

The five intervention groups comprise the two arms of the study. One arm concerns the importance of supplementation using the three supplementation-only groups: carbohydrate supplementation (CARB; 20 g maltodextrin + 10 g sucrose), collagen supplementation (COLL; 20 g bovine collagen protein hydrolysate + 10 g sucrose), and whey supplementation (WHEY; 20 g whey protein hydrolysate + 10 g sucrose). All supplements were developed, prepared, and individually packaged by Arla Foods Ingredients Group P/S, Viby J, Denmark. The other arm investigates the importance of training loads and includes all groups supplemented with the aforementioned whey supplements, either without training (WHEY; the same group used in the supplementation arm), with light load home-based resistance training four times weekly (LITW), or with center-based heavy resistance training three times weekly (HRTW). Table 2 describes the training protocols in detail. All subjects consume the supplements twice daily, namely in the morning and at midday, preferably just before or during meals to increase satiety, thereby avoiding excess intake of calories. Participants who exercise are encouraged to take one supplement immediately after each training session. We conduct all interventions and tests at Bispebjerg Hospital. To report the improvement in thigh strength in repetition maximums (RM), we measure the thigh 3 RM in the knee extensor machine used for heavy resistance training (Super Executive Line; TechnoGym, Cesena, Italy) using as few attempts as possible every third month. To relate to the commonly used 1 RM, we will convert the result to a 1-RM equivalent using the Brzycki formula [60]: 1 RM = weight lifted × [36/(37 − repetitions)].

**Table 2:** Exercise descriptions

Exercise regimen	LITW (three to five times weekly)	HRTW (three times weekly)
General comments	We instruct weekly in the first month and thereafter correct and instruct all exercises monthly, including one home visit. We increase the load accordingly when subjects can perform a given exercise correctly (i.e., optimal range of motion) and steadily (i.e., no shaking and maintaining a steady pace) as evaluated by the instructor. We use red, green, and blue TheraBand® rubber bands (Hygenic Corp., Akron, OH, USA). The weekly number of training sequences varies between three and five as follows: three, four, five, four, three, four, five, four, etc. (i.e., on average, four times weekly).	*Lower body exercises*: All exercises progress from 3 sets of 12 repetitions at 12 RM load to 5 sets of 6 repetitions at 6 RM load in 3-month cycles. The final 2 weeks of each cycle are tapering and testing periods with less intensive training. *Upper body exercises*: All exercises progress from 3 sets of 12 repetitions (12 RM) to 4 sets of 10 repetitions (10 RM).
Leg extension	Sitting on a chair and using rubber bands mounted around one leg of a chair and the relevant ankle, the participant extends and flexes the knee joint continuously and slowly. The exercise is performed three times for 1 minute each, separated by a break of similar duration, where they train the opposite leg. We ensured progress by decreasing the rubber band’s elasticity.	Super Executive Line (TechnoGym, Gambettola, Cesena, Italy). We separate sets by approximately 2 minutes. At all times, loading ensured training to fatigue. If participants could perform more repetitions after the final set, we added more weight at the next session.
Chair-stand/squat/leg press	Without using the arms, participants rise from and sit down on a chair continuously and slowly. They perform the exercise three times for 1.5 minutes separated by breaks of similar duration. We ensured progress by starting with the participant on a high chair, then converting to a low or no chair (i.e., squats).	Super Executive Line (TechnoGym, Gambettola, Cesena, Italy): as described for leg extension
Leg curl	Standing with rubber bands mounted around, for example, the leg of a chair and the participant’s ankle, the participant flexes and extends the knee joint continuously and slowly. The exercise is performed three times for 1 minute separated by breaks of similar duration, where they train the opposite leg. We ensured progress by decreasing the rubber band’s elasticity.	MED Line (TechnoGym, Gambettola, Cesena, Italy): as described for leg extension
Shoulder pull/pull-down	Using rubber bands mounted around, for example, a doorknob, the participant flexes and extends the elbow joint continuously and slowly while keeping elbows shoulder-wide. The participant performs the exercise three times for 1.5 minutes separated by a break of similar duration. We ensured progress by decreasing the rubber band’s elasticity.	TR Equipment model 9014 (Tranås, Sweden): We separated sets by a 2-minute break. At all times, loading ensured training to fatigue. If participants could perform more repetitions after the final set, we added more weight at the next session.
Arm stretch/push-up	Standing against a wall, the participant flexes and extends the elbow joints continuously and slowly. The exercise is performed three times for 1.5 minutes separated by breaks of similar duration. We ensured progress by increasing the distance to the wall, having the participant perform the exercise on the floor while supporting on hands and knees, or performing the exercise on the floor supporting on hands and feet.	TR Equipment model 9025 (Tranås, Sweden): as described for should pulldown

*RM* Repetition maximum

Participants monitor their adherence to protein supplementation and their home-based exercise in hard-copy diaries, whereas the trainers register the training adherence in the HRTW group. We stress the importance of registering only the actual number of supplements taken and exercises performed.

### Blinding

We achieve blinding by storing the randomization log file electronically without access for researchers performing the tests who could be affected by not being blinded. Specifically, blinded radiographers will perform magnetic resonance imaging (MRI) scans at the Department of Radiology, Bispebjerg-Frederiksberg Hospital, Copenhagen, Denmark. A blinded physiotherapist or physiologist will perform strength and functional assessments, and only one tester will assess each participant. Experienced staff not blinded to the intervention will perform dual-energy X-ray absorptiometry (DXA), muscle biopsies, anthropometry, and blood sampling. Blinded investigators will perform all analysis and interpretation of obtained data. We blind participants in supplementation-only groups, as the bags containing supplements are marked only by a code, and only relevant researchers can access the key file containing the explanation of the codes. For obvious reasons, we did not blind those performing exercises to intervention or supplement type, as stated in the study hypotheses.

### Measurements

This section describes each measurement performed to evaluate the longitudinal effects of the chosen interventions. Table 3 displays the time points for each examination. At baseline, we test the peak isometric and isokinetic peak torques as well as leg extensor power in both legs to evaluate if a large side difference between thigh peak torques is associated with an improved or diminished response to the interventions.

**Table 3:** Time schedule of participant engagement

Measurement	Enrollment	Baseline	6 months	12 months	18 months	Other
Enrollment						
Recruitment and screening	X					
Information and consent	X					
Primary outcome						
MRI CSA of quadriceps femoris		X	X	X	X	
Secondary outcomes						
Thigh isometric peak torque		X	X	X	X	
Thigh isokinetic peak torque		X	X	X	X	
Thigh power		X	X	X	X	
30-second chair-stand		X	X	X	X	
Muscle biopsy		X		X		
Body composition (DXA)		X	X	X	X	
Bone mineral density (DXA)		X		X		
Gut microbiome		X	X	X		
Fecal metabolome		X	X	X		
Plasma metabolome		X	X	X		
Acute muscle FSR		X		X		
Tertiary outcomes						
Blood samples		X	X	X	X	
Anthropometry		X	X	X	X	
Hand grip strength		X	X	X	X	
400-m gait speed		X	X	X	X	
SF-36 and PSQI		X		X		
Activity monitoring		X	X	X	X	
Registration of food consumption		X		X		At week 6
Sensory questionnaires			X	X		Weekly for 0–3 months
Satisfaction with Food-related Life scale		X	X	X		
Oral glucose tolerance test		X		X		
Qualitative interview		X			X	
Questionnaire on food habits		X				
Life story interviews						Ad hoc
Ethnographic fieldwork						Ad hoc

*Abbreviations: MRI CSA* magnetic resonance imaging cross-sectional area, *DXA* dual-energy X-ray absorptiometry, *FSR* fractional synthesis rate, *PSQI* Pittsburgh Sleep Quality Index, *SF-36* 36-item Short Form Health Survey

#### Primary outcome

A change in muscle CSA is a direct reflection of the intervention’s ability to restore muscle protein and a direct consequence of the net protein balance over time in the target muscles. We chose m. quadriceps and m. vastus lateralis CSA based on MRI scans of the dominant thigh as the primary outcome, as it directly reflects the intervention’s ability to counteract age-related loss of muscle mass. MRI scans have high sensitivity and validity in terms of lean mass in skeletal muscle [61], and previous researchers have considered this modality superior to DXA [62, 63].

**Fig. 4:**
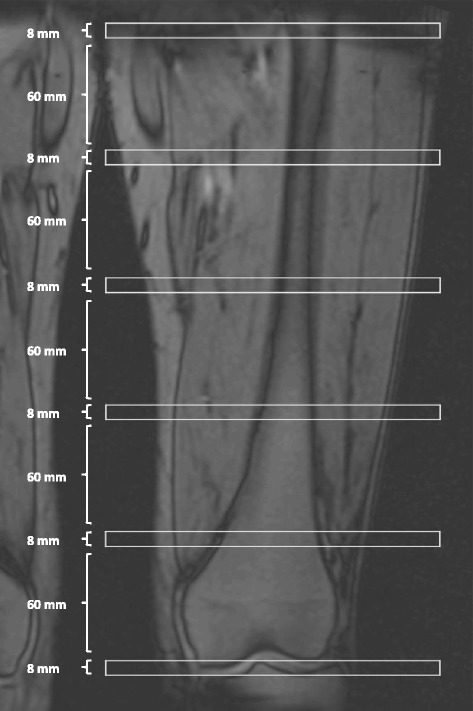
Magnetic resonance imaging analysis. We place slices as shown for analysis of cross-sectional area of the m. quadriceps femoris muscle and analyze slices 3 (counting in distal to proximal direction) and 4 for all subjects, and we use slice 4 for primary outcome evaluation. We fix the placement of slices in absolute distances, but we measure the femur length on dual-energy X-ray absorptiometric scans from the lateral tibial plateau (0 %) to the top of the greater trochanter (100 %) to report the relative placement of slices. Currently, placement of slice 3 ranges from 27 % to 36 % and slice 4 from 40 % to 54 % of the femoral length, depending on the height of the participant

Each scan consists of six axial slices, with the first slice placed in the medial tibia plateau. Each slide is 8 mm thick and separated by a 60-mm gap as shown in Fig. 4. The primary time interval for assessment is from baseline to 12 months.

#### Secondary outcomes

##### Thigh isometric peak torque

After having the participant perform a brief warm-up on a cycle ergometer, a tester will measure the dominant thigh peak strength at 70-degree flexion (0 degrees represents horizontal) in a Kinetic Communicator (model 500-11, Kinetic Communicator; Isokinetic International, Chattanooga, TN, USA) with verbal encouragement. An investigator will choose the best of three sweeps (i.e., highest peak force) for analysis. The rate of force development and impulse will also be analyzed [51, 64].

##### Thigh isokinetic peak torque

Using the same settings as those used for the isometric peak strength exercise, the same tester will measure the dominant thigh isokinetic (at 60 degrees/second) force. With verbal encouragement, participants will perform maximal sweeps until peak values decline markedly from the best sweep on that particular test day, and the test is finished. An investigator will choose the best sweep (i.e., highest peak torque) and analyze the peak torque, total work, and angle at peak torque [51, 64].

##### Power rig

After recording the isometric and isokinetic peak torques, the tester will measure the maximum single-leg extensor power of the dominant leg using a University of Nottingham leg extensor power rig according to procedures described elsewhere [65]. This test is known to correlate with physical performance [8] even more than muscle strength [66] and serves to link the changes in strength to functional outcomes. During the test, participants are in a seated position, and a single maximum explosive leg extension accelerates a flywheel from rest. A computer calculates the power of the leg extensors on the basis of the speed of the flywheel. The participants familiarize themselves with the procedure by performing two warm-up trials followed by a minimum of five and a maximum of ten maximal trials with approximately 30 seconds of rest between them.

##### 30-second chair-stand

The 30-second chair-stand test is a functional assessment of strength and endurance in the lower extremity by measuring the number of stands completed in 30 seconds with hands crossed on the chest [67]. The test is useful in characterizing the participant’s functionality and is also known to correlate with daily activity level [68].

##### Muscle biopsies and fiber-type composition

Working in sterile conditions, a qualified researcher will obtain muscle biopsies from the dominant m. vastus lateralis *ad modum* Bergström [69] with suction using the local anesthetic lidocaine 1 %. One biopsy is taken before and one after 12 months of the intervention, approximately 2–3 cm apart in a proximal-to-distal direction.

##### Body composition

A trained operator will perform whole-body DXA using the enCORE v.16 software (Lunar iDXA; GE Medical Systems, Pewaukee, WI, USA). Participants arrive having refrained from solid foods from 21:00 the day before the baseline and 12-month scans, and we perform scanning between 08:00 and 10:00. We obtain the remaining scans in the fed state at all times of the day, but prior to all scans participants are euhydrated and instructed to void. Whole-body composition is autoanalyzed, and, on a separate occasion, a blinded investigator performs separate thigh analyses manually with a region of interest (ROI) defined proximally by drawing a horizontal line laterally from the distal part of the groin and distally by drawing a line horizontally through the medial tibial plateau [70–72]. The thigh ROIs are equal in size in each subject but vary with thigh length between subjects.

##### Bone mineral density

In the same baseline and 12-month DXA sessions, the same operator scans the bone mineral density in the lumbar region (L2–L4) and the dominant collum femoris. The investigator will place the vertebral ROI as caudal as possible, including the discus caudal to the vertebra, making sure not to include the adjacent vertebra or spinous process. On the collum femoris scan, the investigator places the ROI as distal as possible without including the greater trochanter [73, 74].

##### Gut microbiome

At 0, 6, and 12 months, participants place a fecal sample in an insulated bag with freezer elements until delivery at Bispebjerg Hospital within 48 h. The container is then stored at −60 °C until further analysis. Following homogenization, we extract the total DNA using the PowerSoil DNA isolation kit (MO BIO Laboratories, Carlsbad, CA, USA) with an initial bead-beating step and employ tag-encoded *16S* ribosomal RNA gene (prokaryotes) and internal transcribed spacer region (eukaryotes) high-throughput sequencing using the MiSeq platform (Illumina, San Diego, CA, USA) to characterize the prokaryotic and eukaryotic components of the gut microbiome. As previously reported [75], we purify, extract, and sequence the virus-like particles for characterization of the gut virome.

##### Fecal metabolome

We will analyze the fecal metabolite extracts by performing gas chromatography with time-of-flight mass spectrometry (GC-TOF-MS) and nuclear magnetic resonance (NMR) spectroscopy using the same metabolite extract. The extraction procedure will involve stabilization of the homogenized fecal samples in PBS (pH 7.4) followed by freeze-drying and subsequent resuspension in methanol. We will perform untargeted metabolomics analysis and targeted profiling of short-chain fatty acids with a high-throughput GC-TOF-MS setup consisting of an Agilent 7890B gas chromatograph (Agilent Technologies, Santa Clara, CA, USA) and a high-throughput Pegasus TOF-MS spectrophotometer (LECO, St. Joseph, MI, USA). To increase GC-TOF-MS sensitivity toward a broad spectrum of nonvolatile metabolites, we will derivatize the samples as previously described [76] followed by processing of the obtained complex raw data using state-of-the-art three-way decomposition methods with Parallel Factor Analysis 2 [77]. We will further stabilize the fecal metabolite extracts using PBS prior to performing one-dimensional ^1^H NMR measurements using an AVANCE III 600 spectrometer (Bruker BioSpin GmbH, Rheinstetten, Germany) operating at a Larmor frequency of 600.13 MHz for protons and equipped with a cooled high-capacity autosampler (SampleJet; Bruker BioSpin GmbH) [78].

##### Plasma metabolome

We will collect the plasma samples with participants in fasting condition prior to the oral glucose tolerance test (OGTT) at 0 and 12 months and in fed condition at 6 and 18 months. Researchers will draw the blood into two containers, one containing tripotassium ethylenediaminetetraacetic acid (K_3_-EDTA) and one with heparin, and, after centrifugation at 3970 rpm for 10 minutes at 4 °C, we will pipette plasma into vials that are stored at −60 °C. We then will perform untargeted metabolomics of all samples using proton one-dimensional ^1^H NMR spectroscopy and for a subset of the samples by GC-TOF-MS.

We will perform the NMR measurements according to standard operating procedures developed for NMR metabolomics of plasma samples [79], and the data will provide unbiased metabolic fingerprints of the plasma samples, including information about lipoprotein particle distribution [80]. Prior to GC-TOF-MS analysis, we will thaw, vortex, and centrifuge the plasma samples at room temperature followed by addition of ice-cold acetonitrile in 1:3 (vol:vol) to precipitate proteins. Immediately after this, we will vortex the samples vigorously and centrifuge them at 20,000 × *g* for 10 minutes at 4 °C, and we will then dry 50 μl of clear supernatant under reduced pressure, derivatize it as described previously [76], and subject it to GC-TOF-MS.

##### Muscle protein synthesis

To assess MPS, we will perform an 8.5-h primed, continuous infusion of a stable isotope-labeled amino acid tracer, L-[ring-^13^C_6_]phenylalanine (Cambridge Isotope Laboratories, Tewksbury, MA, USA) at baseline and after 12 months of the intervention in a random subset of participants from each of the five intervention groups. The participants will fast overnight fast, and we will start the tracer infusion followed by collection of muscle biopsies after 1.5-h and 4.5-h infusions to measure the basal resting MPS over a 3-h period. Thereafter, we provide a protein drink as a beverage containing 20 g of whey hydrolysate plus 10 g of maltodextrin followed by collection of the third muscle biopsy. After an additional 4 h, we will measure the postprandial response of MPS. We will sample the muscle biopsies from the m. vastus lateralis muscle approximately 3 cm apart in a proximal-to-distal direction. Throughout the day, we will collect venous blood samples to measure tracer enrichment as well as amino acid and insulin concentrations, and we will measure the enrichment of phenylalanine tracer in blood and free in muscle cells by GC-MS/MS, whereas we will measure the incorporation of labeled phenylalanine in muscle proteins using GC-combustion/isotope ratio MS. We will then calculate the fractional synthesis rate (FSR) as the difference of incorporated tracer between subsequent biopsies (E_p2_ − E_p1_) divided by the precursor enrichment (E_precursor_; venous plasma and muscle free enrichment) and divided by incorporation time: FSR (%/h) = [(E_p2_ − E_p1_) × (E_precursor_)^−1^ × (incorporation time)^−1^] × 100 % [81]. This setup allows the evaluation of both basal MPS and the MPS response to a protein/carbohydrate drink before and after the 12-month intervention as a measure of muscle anabolic responsiveness to intake of a single protein supplement. We will also analyze muscle biopsies for relevant myocellular gene expression using real-time reverse transcriptase-polymerase chain reaction and for protein signaling through, for example, mammalian/mechanistic target of rapamycin complex 1 by performing Western blotting.

#### Tertiary outcomes

##### Blood parameters and anthropometry

At baseline, we will obtain standard health screening blood samples. At the remaining time points, we will measure only plasma HbA1c, cholesterol, and creatinine. Moreover, we will measure body weight, waist, and hip circumference at the remaining time points, whereas we will measure only height at baseline. Participants will wear underwear only when we measure body weight at baseline and 12 months, and they will wear light clothing at 6 and 18 months.

##### Hand grip strength

We will evaluate hand grip strength of the dominant hand (bilateral testing at baseline) by using a hand grip strength dynamometer (DHD-1 [SH1001]; SAEHAN Corporation, Changwon City, South Korea). The tester allows at least 30 seconds of rest between a minimum of three attempts, and the highest value at the given time point is used. The test is finished when two consecutive measurements are lower than the peak value [82].

##### 400-m walk test (fast gait speed)

On a 20-m course marked with two colored cones, the physical examiner instructs the subject to walk 400 m as fast as possible without personal support or sitting down. Up to 1 minute of standing is permitted if the participant feels tired or experiences discomfort, as long as the test is completed within 15 minutes [83]. Among several functional measures, we chose 400-m gait speed as it is one of the more demanding functional tests available for elderly populations. This minimizes the risk of a ceiling effect among the included participants whom we expected to be relatively well-functioning.

##### Health-related questionnaires

At baseline and 12 months, we will hand out Danish translations of the 36-item Short Form Health Survey [84] and the Pittsburgh Sleep Quality Index [85], which the subjects complete without further instructions.

##### Activity monitoring

We measure 4-day (96-h) overall activity by mounting an activity monitor (activPal 3™, activPal 3c™, or activPal micro; PAL Technologies, Glasgow, UK) on the anterior surface of the thigh. The period always covers an entire weekend. An investigator will analyze data quantified as step counts, time in sitting/lying position, time in standing position, and time walking [86, 87].

##### Weighed food registration

We will administer a 3-day weighed food and liquid registration from Wednesday to Friday before, 4–6 weeks after, and 50 weeks after commencing the intervention. Investigators subsequently will quantify total daily intake and meal distribution of macronutrients using the MADLOG VITA system (MADLOG ApS, Kolding, Denmark).

##### Sensory and hedonic evaluation questionnaires

Participants will complete a questionnaire about their experience with taking the dietary supplements. We will collect baseline data on the first consumption day (day 0) and weekly for 12 weeks. Thereafter, we will reevaluate participants every third month until week 52. The flavor of the supplements will be alternated every week between fruit and cacao, and the participants will fill in the questionnaires within 1 minute after ingestion of the supplement. We will measure the participants’ acceptance after the first sip and after complete consumption on a 9-point hedonic scale ranging from 1 = “do not like at all” to 9 = “like a lot,” with neutral in the middle. After the first sip, we will record the participants’ perceived experiences using the check-all-that-apply method with 20 attributes of taste, flavor, mouth feel, and sensory appeal of the dietary supplement. After participants consume the supplements, we will collect data on how satiating, refreshing, and easy to drink it was, as well as on the strength and persistence of the aftertaste on a 9-point scale ranging from 1 = “completely disagree” to 9 = “completely agree,” with neutral in the middle.

##### Satisfaction with Food-related Life scale

Subjects will complete the Satisfaction with Food-related Life scale [88] on days 0, 90, 180, and 360 to identify factors contributing to satisfaction with food-related lifestyle.

##### Oral glucose tolerance test

Participants will undergo two OGTTs during the study. Participants will fast overnight, and a researcher will draw a basal venous blood sample and then administer 75 g of anhydrous glucose dissolved in 250 ml of tap water. Subsequently, the researcher will draw blood at 45 and 120 minutes after consumption of the glucose. Participants will lie supine throughout the 2-h period [89]. We will draw blood in K_3_-EDTA vials and cool the vials for at least 15 minutes, centrifuge the sample for 10 minutes at 3970 rpm at 4 °C, immediately analyze the plasma for glucose, and store aliquots at −80 °C for subsequent insulin measurement. At baseline, we draw two further blood samples in K_3_-EDTA and heparin vials respectively, which are cooled on ice, centrifuged for 10 minutes at 3172 × *g*, and the plasma is then stored at −60 °C until metabolome analysis.

#### Qualitative interviews and observations

##### Qualitative interviews

Upon participant inclusion, a researcher will conduct a short, standardized, qualitative interview with the participant to gain information on marital status, living conditions, work life, hobbies, and dietary preferences. At the 18-month follow-up, a researcher will interview the participants to gain information about their experience with the intervention and current habits regarding nutrition and exercise.

##### Questionnaire on food habits

At baseline, the staff will hand out a questionnaire with a range of questions about the food perceptions and habits of the participant. The questionnaire combines basic socioeducational data, quantitative questions, and quantifiable qualitative questions about lifestyle changes, dietary changes and perceptions, and intake of protein-rich foods. The participants can complete the questionnaires during the first 3 months and subsequently hand them in.

##### Life story interviews

We will select 25 of the intervention participants for 1.5- to 2.5-h life story/trajectory interviews in their own homes. The main themes of the interviews are past and present perceptions of and habits concerning food and physical activity, but we will also include questions regarding mobility and work life.

##### Ethnographic fieldwork

We will select 48 research participants to engage in a qualitative study focused on everyday life routines, eating practices, physical activity, and experiences with participating in the trial. We do this by observing the research participants in their homes and at the site of the clinical trial, thereby following their daily activities in both settings. Further, we will conduct semistructured qualitative interviews to gain in-depth knowledge about selected individuals.

### Data collection, management, and analysis

According to Danish research ethics legislation, we will inform the participants about their rights as voluntary subjects in a scientific trial and interview them about their motivation for participation. We do this to make participants consider participation thoroughly to diminish the likelihood of their dropping out. We are obliged to report any unforeseen adverse effects due to participation to the local ethical committee, which will consider terminating the project depending on the reported incidents.

We will collect and manage study data using REDCap electronic data capture tools hosted at RegionH (Copenhagen, Denmark) [90] and secure it according to Danish Data Protection Agency legislation (approval on 9 January 2015, number 2012-58-0004 – BBH-2015-001 I-Suite 03432). This includes pseudoanonymization of sensitive data. Only relevant staff have logged access to the key file.

The project has a scientific advisory board with experts from all scientific disciplines applied in the research, and its members meet annually with the researchers to discuss progress and plan future work. The board members are financially and personally unrelated to the study investigators and the project as such.

The principal investigators from each scientific discipline have a shared responsibility to secure and monitor data collection and interpretation, and thus they are all involved in project management, analysis of samples, data collection, and observations and will all have access to the final dataset and jointly be involved in the interpretation of results.

### Statistics and power calculations

For all power calculations, we will apply a level of significance of 0.05 and a power of 0.80, and the primary outcome is the degree of muscle hypertrophy determined as the change in quadriceps muscle CSA on MRI scans over the course of the 12-month intervention. On the basis of previous data [91], we set the following parameters: least average expected change over the course of the 12-month intervention between distinct intervention groups, 300 mm^2^ (corresponding to an estimated relative change of 6.5 %) with a SD set to 140 mm^2^. Our goal is to be able to detect differences between groups’ mean hypertrophy down to approximately 2 %, which corresponds to approximately 80 mm^2^. A difference in the change of quadriceps muscle CSA greater than 80 mm^2^ between any two groups is statistically detectable with a power of at least 0.80 with a group size of 30. We decided to include 35 subjects in each of the three groups (HRTW, LITW, and CARB), as we anticipated a dropout rate of 15 % at the time of study initiation. In the WHEY and COLL groups, we expected a larger dropout rate because of taste issues with protein supplements and no motivational effect by training as in the HRTW and LITW groups. Thus, we will include 50 each in the WHEY and COLL groups. On an ad hoc basis, we will evaluate the actual dropout rate and adjust the total number of inclusions accordingly.

In a subset of participants from the five intervention groups, we will conduct experimental tracer studies before and after the 12-month intervention period to determine the extent to which the interventions affect the responsiveness of MPS to protein intake. The goal is to measure whether any of the interventions enhance the anabolic sensitivity to protein intake and thereby investigate if we can link the observed differences in muscle mass over the course of the intervention period to changes in the anabolic response to protein feeding. Thus, the outcome measure is the relative change in the FSR of muscle proteins from the basal condition, defined as the overnight fasted and resting state, to the average 4-h postprandial period after intake of a standard protein bolus. We are not aware of any data on changes in MPS responsiveness over time, and we therefore based the power calculations on a sum of our own published and unpublished data obtained in healthy elderly participants. We established the following parameters: expected average 4-h postprandial responsiveness to protein feeding from basal condition of 0.016 %/h (i.e., approximately corresponding to an increase in muscle protein FSR of 50 %) with an interparticipant SD on the increase of 0.017 %/h. We aim to be able to detect a 50 % change in responsiveness within a group over the course of the 12-month period (i.e., a change of 0.008 %/h in the increase in the muscle protein FSR from 0.016 %/h to 0.024 %/h). The SD for the change in responsiveness is set as 0.006 %/h. By including 10–12 participants, we obtain a power of at least 0.80 to detect a statistical difference over time within each intervention group. By taking the probability of dropouts into account, we decided to include 12 participants in the HRTW, LITW, and CARB groups and 15 in the WHEY and COLL groups, respectively.

Investigators will analyze data as intention-to-treat (except in subgroup analyses) as well as per protocol, and we will test the following groups against each other using one-way analysis of variance of the relative group changes from baseline to 12 months: (1) WHEY vs. COLL vs. CARB and (2) HRTW vs. LITW vs. WHEY. We will not conduct any interim analyses in this project.

## Discussion

The inclusion rate is currently 15 %, and we have been in contact with more than 1000 elderly individuals, recruiting approximately 150 participants. The major challenge until now has been to find elderly individuals who are willing to be committed to participate for 18 months as well as accept the outcome of the randomization. The interventions are very different in terms of time required for the intervention as well as potential physical and social gain. The three supplementation-only groups demand less time, but the candidates expect less gain from the intervention than those in the more time-consuming physical training groups, so the main reasons for candidates to decline participation is the uncertainty of the randomization. This has resulted in roughly 45 % of candidates having decided to refrain from volunteering. Second, our exclusion criteria are numerous in order to ensure accurate results, and we have excluded around 40 % of the potential participant population for this reason, leaving for inclusion 15 % of those who had initially contacted us. The invasive procedures and number of tests are not issues of major importance for the participants when we are recruiting. Fortunately, the dropout rate until now has been very low: Only 4.3 % have ceased participation, which is far less than the initially expected rate of 10–15 %. We recruit research participants for this study in the Capital Region of Denmark. The population in this region is wealthier [92], and likely also healthier, than the general population in Denmark, but for practical reasons we could only recruit participants locally. This represents a possible selection bias in the trial.

### Safety issues and dropout rate

We consider the project safe, and the clinical staff routinely perform the tests and invasive procedures used. We supervise participants during heavy strength training and carefully instruct them in the light load training exercises. The nutrient supplements contain 510 kJ (i.e., a dose of 1020 kJ/day) and induce mild satiety when ingested. Therefore, we expect a slight decrease in the participants’ habitual diet corresponding to the energy content in the supplements, and we expect their body weight to be stable. We control HbA1c, creatinine, and cholesterol levels after 6 months to ensure that participants do not develop insulin resistance, impaired kidney function, or hyperlipidemia because of supplement intake. In case of adverse events, the Danish Patient Compensation Association covers participants’ treatment needs.

### Perspectives

The purpose of the CALM Intervention Study is to provide interdisciplinary knowledge on the efficacy and feasibility of daily protein supplementation and muscular exercise training in different settings to support the maintenance of skeletal muscle mass in the elderly to ensure daily functional capacity and maintain or improve quality of life. Until now, only a few studies have investigated the physiological outcome of strength training and protein supplementation, and, to our knowledge, a light intensity strength training regime has never been investigated. Further, the interdisciplinary setting, including sensory, fecal, and qualitative investigations, will provide unique insights into how to counteract the age-related loss of skeletal muscle mass.

A key assumption in the CALM Intervention Study is that an improved adherence to a healthier lifestyle requires sensitivity to the everyday lives, routines, and cultural differences of the research participants [93]. We aim to enhance this sensitivity by gathering qualitative knowledge on the societal, cultural, and individual factors that influence daily routines related to nutrient consumption and exercise to identify and circumvent the barriers that complicate changes in these. We will perform an exploration of societal and individual influences on everyday lifestyle habits and of how the project configures the participants’ everyday food and exercise practices. Further, we will investigate how participants’ practices are (and are not) transformed by their interactions with the materiality as well as the scientific practitioners and attitudes of the project to provide insights that potentially could improve health promotion strategies [94].

We expect to translate the results of this into guidelines targeted to the home-dwelling, independent elderly population and disseminate them to the public through channels involving nongovernmental organizations, newspapers, electronic media, and stakeholders in, for example, the municipalities. The CALM Intervention Study will provide novel information on the potential longitudinal benefits of protein supplementation and strength training regarding physical performance, gut microbiota, and overall health while taking into account the feasibility and impact of the interventions on everyday life.

### Trial status

Since we began advertising for participants in January 2014, we have recruited and randomized 153 (as of 1 August 2016). A total of 54 and 32 participants have completed the intervention and follow-up, respectively.

## Abbreviations

CALM, Counteracting Age-related Loss of Skeletal Muscle Mass; CARB, carbohydrate supplementation group; COLL, collagen supplementation group; COPD, chronic obstructive pulmonary disease; CSA, cross-sectional area; DXA, dual-energy X-ray absorptiometry; EULAR, European League Against Rheumatism; FEV_1_, forced expiratory volume in 1 second; FSR, fractional synthesis rate; FVC, forced vital capacity; GC-TOF-MS, gas chromatography with time-of-flight mass spectrometry; GOLD, Global Initiative for Chronic Obstructive Lung Disease; HRTW, heavy resistance training with whey supplementation; ITS, internal transcribed spacer; K_3_-EDTA, tripotassium ethylenediaminetetraacetic acid; LITW, light-intensity training with whey supplementation; LVEF, left ventricular ejection fraction; MPS, muscle protein synthesis; MRI, magnetic resonance imaging; NMR, nuclear magnetic resonance; OGTT, oral glucose tolerance test; PDCAAS, protein digestibility-corrected amino acid score; PSQI, Pittsburgh Sleep Quality Index; RCT, randomized controlled trial; RM, repetition maximum; ROI, region of interest; SF-36, 36-item Short Form Health Survey; SPIRIT, Standard Protocol Items: Recommendations for Interventional Trials; SWFL, Satisfaction with Food-related Life scale; WHEY, whey supplementation
